# Two new species of the *Fusarium fujikuroi* species complex isolated from the natural environment

**DOI:** 10.1007/s10482-017-0855-1

**Published:** 2017-03-16

**Authors:** Tarek A. A. Moussa, Hassan S. Al-Zahrani, Naif M. S. Kadasa, Sarah A. Ahmed, G. Sybren de Hoog, Abdullah M. S. Al-Hatmi

**Affiliations:** 1 Biological Sciences Department, Faculty of Science, King Abdulaziz University, Jeddah, Saudi Arabia; 2 Biological Sciences Department, Faculty of Science, University of Jeddah, Jeddah, Saudi Arabia; 3 Westerdijk Fungal Biodiversity Institute, PO Box 85167, 3508 AD Utrecht, The Netherlands; 4 Botany and Microbiology Department, Faculty of Science, Cairo University, Giza, Egypt; 5 Department of Medical Microbiology, Faculty of Medical Laboratory Sciences, University of Khartoum, Khartoum, Sudan; 6 Institute of Biodiversity and Ecosystem Dynamics, University of Amsterdam, Amsterdam, The Netherlands; 7 Directorate General of Health Services, Ministry of Health, Ibri Hospital, Ibri, Oman

**Keywords:** *Fusarium*, Saprobe, Morphology, Molecular phylogeny

## Abstract

Two new species in the *Fusarium fujikuroi* species complex (FFSC) are introduced. One of these, represented by strain CBS 454.97 was isolated from plant debris (*Striga hermonthica*) in the Sudan, while the second, represented by strains CBS 119850 and CBS 483.94, which originated from soil in Australia. Molecular analyses were performed including *TEF1* spanning 576 bp region, 860 bp region of *rPB2*, and 500 bp *BT2* region. Phylogenetic trees based on these regions showed that the two species are clearly distinct from all known taxa in the *F. fujikuroi* species complex. Based on phenotypic, physiological characters and molecular data, we introduce *Fusarium sudanense* and *Fusarium terricola* as novel species in the complex.

## Introduction

*Fusarium* is a large and variable genus with nearly 300 recognized species occurring worldwide in a diversity of habitats. Particularly in plant pathology, species have extensively been studied because of their opportunism on numerous hosts, among which are economically important crops. For example, many *formae speciales* have been reported in *F. oxysporum* and relatives (Ordonez et al. 2015) as etiologic agents of plant diseases. Some species seem to have a narrow host range or may even be host-specific, such as *Fusarium ficicrescens* that has as yet only been found on figs (Al-Hatmi et al. 2016a). Members of the genus are increasingly observed as agents of human infection (Al-Hatmi et al. 2016b). A further significant property is their production of mycotoxins, especially in *Fusarium* species that occur in association with farm animals receiving cereal-based diets (de Nijs et al. 1997).

Typically, most species are soil-borne, causing diseases in seedlings or weakened plants (Watanabe 2013). *Fusarium* is a common mould in the environment and different environmental factors, such as moisture, temperature, nutrients and other ones appear to be of great importance for colonization of a wide diversity of substrates and ecological niches (Smith 2007). Geographical factors including climate are of prime importance for the diversity of *Fusarium* species (Summerell et al. 2010; Karim et al. 2016). Strictly saprobic *Fusarium* have received less attention, though they are widely distributed in natural habitats, notably in soil, where they might have a role in the turnover of organic matter (Karim et al. 2016). However, saprobic strains may become opportunistic upon availability of a susceptible host (Rep et al. 2005). Furthermore, given the widespread occurrence of *Fusarium* in the environment, it seems reasonable to hypothesize also that pathogenic forms of *Fusarium* may have evolved from non-pathogenic ancestors (Alves-Santos et al. 1999). Thus, many *Fusarium* species with importance to environment, agriculture and human health have a reservoir in soil, and their infections in a wide range of plants (Wakelin et al. 2008), animals (O’Donnell et al. 2016) and humans (Al-Hatmi et al. 2016b) are regarded to be of an opportunistic nature.

The *Fusarium fujikuroi* species complex (FFSC) is one of the larger groups within the genus *Fusarium* with various ecologies (Nirenberg and O’Donnell 1998; O’Donnell et al. 2000; Al-Hatmi et al. 2015). Studies suggested that with the use of molecular data more than 50 phylogenetic species within the *fujikuroi* complex might be recognized (O’Donnell et al. 2015). Recently, Herron et al. (2015) described eight more species in the *fujikuroi* complex from stem cankers and branches of *Pinus* plants. Laurence et al. (2015) added three additional species from Australian natural forests, Al-Hatmi et al. (2016a) described *F. ficicrescens* from figs in Iran and Edwards et al. (2016) published *F. agapanthi* as a novel plant pathogen from Australia and Italy.

Recent and historical ecosystem surveillance in Australia has resulted in the discovery of novel *Fusarium* species including *F. aywerte, F. babinda*, *F. beomiforme*, *F. burgessii*, *F. coicis*, *F. gaditjirri*, *F. goolgardi*, *F. lyarnte*, *F. mundagurra*, *F. nurragi*, *F. newnesense*, *F. nygamai*, *F. tjaetaba, F. tjaynera* and *F. werrikimbe* (Laurence et al. 2015). This number has increased to 16 species with the recent description of *F. agapanthi* above (Edwards et al. 2016). In the present study, the taxonomic status of all available strains of the *F. fujikuroi* species complex was verified using a polyphasic approach. The resultant data show that some isolates represent two new *Fusarium* species, for which we propose the names *Fusarium terricola* for a species isolated from Australia and *Fusarium sudanense* that was isolated in Sudan.

## Materials and methods

### Strains

Three strains in the reference collection of Centraalbueau voor Schimmelcultures (housed at Westerdijk Fungal Biodiversity Institute), previously identified morphologically as *F. nygamai*, were analyzed and compared with all available members of the *F. fujikuroi* species complex. Two of these strains (CBS 119850 and CBS 483.94) were isolated from soil in Australia, while an additional strain (CBS 454.97) originated from *Striga hermonthica*. The latter strain was included in a multilocus molecular phylogenetic analysis as *Fusarium* sp. NRRL 26793 as a distinct clade (Herron et al. 2015; Laurence et al. 2015).

### Morphology

Colony characteristics and growth morphology were studied by inoculating the isolates onto plates of Malt Extract Agar (MEA; Oxoid, U.K.), Oatmeal Agar (OA; home-made at CBS), Potato Dextrose Agar (PDA; Oxoid), Synthetic Nutrient Agar (SNA; CBS) (Nirenberg 1976) and carnation leaf agar (CLA; CBS) (Leslie and Summerell 2006). Cultures were grown under 12 h light–dark (l/d) cycles with UV and daylight colour fluorescent lights at 24 °C. Morphological characters examined included the shape and size of macroconidia produced in sporodochia on Carnation Leaf Agar (CLA) (Fisher et al. 1982), the shape and mode of formation of microconidia on CLA and SNA (Nirenberg 1976), the production of chlamydospores on CLA, and pigmentation of the agar on Potato Dextrose Agar (PDA). Microscopic slides were prepared for each isolate by mounting structures in lactic acid and the slides were made from cultures grown on CLA plates which were observed after 5 days of incubation at 24 °C. Slides were examined with a Nikon Eclipse 80i light microscope, and pictures were taken using a camera attached to the microscope (Nikon; digital-sight DS-5M). A minimum of 10 measurements per structure were taken and the average was calculated.

### Growth rate

Cardinal growth temperatures were determined on MEA and PDA plates incubated in the dark for 2 weeks at temperatures of 18–40 °C at intervals of 3 °C; with two replicates for each isolate. Average growth rates per species were calculated and expressed as diametric growth per 24 h.

### DNA amplification and sequencing

The following partial genes were amplified directly from genomic DNA for multilocus sequence typing: elongation factor 1 alpha (*TEF1*) (O’Donnell et al. 2010), the second largest subunit of RNA polymerase (*rPB2*) (Reeb et al. 2004), and β-tubulin (*BT2*). PCR amplification and sequencing were performed according to the protocol applied by Al-Hatmi et al. (2016a).

### Phylogenetic inference

To confirm the identity of our presumed new *Fusarium* species, we evaluated their position in Bayesian phylogenetic and RAxML trees of the following individual gene markers (*BT2*, *TEF1* and *rPB2*). In these analyses, our sequences, together with sequences retrieved from GenBank were analysed (Table 1). Sequences were aligned with MAFFT (www.ebi.ac.uk/Tools/msa/mafft/), followed by manual adjustments with MEGA v6.2 and BioEdit v7.0.5.2. A single alignment was constructed for *TEF1* and *BT2* and *rPB2*. The analysis included 58 sequences for *TEF1*, 50 sequences for *BT2* and 32 sequences for *rPB2*. The best-fit model of evolution, determined by MEGA v6.2, was used to infer the appropriate substitution model that would best fit the model of DNA evolution for each sequence data set. Maximum likelihood (ML) and Bayesian inference (BI) analyses were used to estimate phylogenetic relationships. ML analysis was performed with RAxML-hpc v7.0.3 (Stamatakis et al. 2005; Stamatakis 2006) with a K2+G model of evolution for *TEF1, BT*, *rPB2* and the combined data. Nodal support was determined by nonparametric bootstrapping (BS) with 1000 replicates. BI analysis was performed in a likelihood framework as implemented in mrbayes v3.0b4 to reconstruct phylogenetic trees (Huelsenbeck and Ronquist 2001). Multiple Bayesian searches using Metropolis-coupled Markov chain Monte Carlo sampling were conducted. One cold and three heated Markov chains were used in the analysis. Analyses were run for 10 million generations, with trees sampled every 1000 generations. The first 25% of the trees, which represented the burn-in phase of the analysis, were discarded. The remaining trees were used for calculating posterior probabilities (PP) of recovered branches (Larget and Simon 1999) in the 50% majority rule consensus tree. Sequences included in this study were supplemented with those from GenBank *Fusarium oxysporum* was used as outgroup and the GenBank accession numbers for the three strains are shown in Table 1.

**Table 1:** GenBank accession numbers of the *F. fujikuroi* species complex used in phylogenetic analysis of *F. terricola* and *F. sudanense*

Species	Collection	β-tubulin	TEF1α	RPB2	Reference
*F. acutatum*	NRRL 13308	U34431	AF160276	(CBS402.97)/KT154005	Scauflaire et al. (2011), Al-Hatmi et al. (2016a)
*F. agapanthi*	NRRL 54465	KU9006361	KU9006311	KU9006261	Edwards et al. (2016)
*F. andiyazi*	CBS 119857	KP662894	KP662901	CBS 119857/KT154004	Al-Hatmi et al. (2016a)
*F. anthophilum*	NRRL 13602	U61541	AF160292	(CBS222.76)/KT154006	Scauflaire et al. (2011), Al-Hatmi et al. (2016a)
*F. bactridioides*	NRRL 20476	U34434	AF160290	–	Scauflaire et al. (2011)
*F. begoniae*	NRRL 25300	U61543	AF160293	–	Scauflaire et al. (2011)
*F. brevicatenulatum*	NRRL 25446	U61623.1	AF160265	–	Scauflaire et al. (2011)
*F. bulbicola*	NRRL 13618	U61546	AF160294	KF466404	Scauflaire et al. (2011), Proctor et al. (2013)
*F. circinatum*	NRRL 25331	U61547	AF160295	JX171623	Scauflaire et al. (2011), O’Donnell et al. (2013)
*F. coicis*	RBG 5368	–	KP083251	KP083274	Laurence et al. (2015)
*F. concentricum*	NRRL 25181	U61548	AF160282	–	Scauflaire et al. (2011)
*F. denticulatum*	NRRL 25302	U34453.1	AF160271	–	Scauflaire et al. (2011)
*F. dlaminii*	NRRL 13164	U34430	AF160277	–	Scauflaire et al. (2011)
*F. ficicrescens*	CBS 125178	KP662896	KP662899	KT154002	Al-Hatmi et al. (2016a)
*F. fracticaudum*	CMW: 25245	KJ541051	KJ541059	–	Herron et al. (2015)
*F. fractiflexum*	NRRL 28852	AF160315	AF160288	–	Scauflaire et al. (2011)
*F. fujikuroi*	NRRL 13566	U34415	AF160279	EF470116	Scauflaire et al. (2011), O’Donnell et al. (2007)
*F. globosum*	NRRL 26131	U61557	AF160285	KF466406	Scauflaire et al. (2011), Proctor et al. (2013)
*F. guttiforme*	NRRL 22945	U34420	AF160297	JX171618	Scauflaire et al. (2011), O’Donnell et al. (2013)
*F. inflexum*	NRRL 20433	U334435	AF8479	JX171583	Scauflaire et al. (2011), O’Donnell et al. (2013)
*F. konzum*	MRC 8544	EU220234	EU220235	–	Scauflaire et al. (2011)
*F. lactis*	NRRL 25200	U61629	AF160272	KM582794	Scauflaire et al. (2011), Triest et al. (2015)
*F. mangiferae*	NRRL 25226	U61561	AF160281	JX171622	Scauflaire et al. (2011), O’Donnell et al. (2013)
*F. marasasianum*	CMW: 25261	KJ541054	KJ541063	–	Herron et al. (2015)
*F. mudagurra*	RBG 5717	–	KP0832561	KP0832761	Laurence et al. (2015)
*F. musae*	NRRL 28893	FN545374	FN552092	FN552114	Van Hove et al. (2011)
*F. napiforme*	NRRL 13604	U34428	AF160266	EF470117	Scauflaire et al. (2011), O’Donnell et al. (2007)
*F. nygamai*	NRRL 13448	U34426	AF160273	EF470114	Scauflaire et al. (2011), O’Donnell et al. (2007)
*F. parvisorum*	CMW: 25267	KJ541055	KJ541060	–	Herron et al. (2015)
*F. pininemorale*	CMW: 25243	KJ541049	KJ541064	–	Herron et al. (2015)
*F. phyllophilum*	NRRL 13617	U34432	AF160274	KF466410	Scauflaire et al. (2011), Proctor et al. (2013)
*F. proliferatum*	NRRL 22944	U34416	AF160280	JX171617	Scauflaire et al. (2011), O’Donnell et al. (2013)
*F. pseudoanthophilum*	NRRL 2520	U61631	AF160264	–	Scauflaire et al. (2011)
*F. pseudocircinatum*	NRRL 22946	U34453	AF160271	–	Scauflaire et al. (2011)
*F. pseudonygamai*	NRRL 13592	U34421	AF160263	–	Scauflaire et al. (2011)
*F. ramigenum*	NRRL 25208	U61632	AF160267	KF4664121	Scauflaire et al. (2011)
*F. sacchari*	NRRL 13999	U34414	AF160278	JX171580	Scauflaire et al. (2011), O’Donnell et al. (2013)
*F. sororula*	CMW: 40578	KJ541057	KJ541067	–	Herron et al. (2015)
*F. subglutinans*	NRRL 22016	U34417	AF160289	JX171599	Scauflaire et al. (2011), O’Donnell et al. (2013)
*F. succisae*	NRRL 13613	U34419	AF160291	–	Scauflaire et al. (2011)
*F. sudanense*	CBS 454.97	KU603909	KU711697	KU604266	This study
*F. sterilihyphosum*	CML 283	DQ445780	DQ452858	–	Scauflaire et al. (2011)
*F. temperatum*	MUCL 52436	HM067692	HM067684	–	Scauflaire et al. (2011)
*F. terricola*	CBS 483.94	KU603908	KU711698	KU604267	This study
*F. terricola*	CBS 119850	KU603907	KU711699	KU604268	This study
*F. tjaetaba*	RBG 5361	–	KP083263	KP083275	Laurence et al. (2015)
*F. thapsinum*	NRRL 22045	U34444	AF160270	JX171600	Scauflaire et al. (2011), O’Donnell et al. (2013)
*F. udum*	NRRL 22949	U34433	AF160275	–	Scauflaire et al. (2011)
*F. verticillioides*	NRRL 22172	U34413	AF160262	EF470122	Scauflaire et al. (2011), O’Donnell et al. (2013)
*Fusarium* sp.	NRRL 26756	–	AF1603071	–	O’Donnell et al. (2000)

## Results

Using the BLAST similarity search (performed on January 15 2017), the *TEF1* region of the strain CBS 454.97 showed 99% (546/547 bp) similarity to *F. andiyazi* strain F16 (JX307409.1) which appears to be wrongly labeled in GenBank. Another closely related strain was *Fusarium* sp. NRRL 26793 with 99% similarity. Further comparison using the FUSARIUM ID database (http://isolate.fusariumdb.org) (Geiser et al. 2004) revealed *Gibberella fujikuroi* species complex (GFSC) NRRL 26793 with 99.83% identity, while the *Fusarium* MLST database (http://www.cbs.knaw.nl/fusarium) (O’Donnell et al. 2010) yielded *F. nygamai* with 99.82% similar to NRRL 26793 (AF160309). CBS 119850 and CBS 483.94 showed a similarity of 100% with *F. andiyazi* strain F16 (JX307409.1) in GenBank, *G. fujikuroi* species complex (GFSC) with 98.93% similarity in FUSARIUM ID, and NRRL 26793 *Fusarium* sp. with 98.9% similarity in *Fusarium* MLST.

Using a BLAST similarity search, the *rPB2* region of strain CBS 454.97 (KU604266) showed 99% (791/794 bp) similarity to *F. nygamai* (FRC M-7492 = KF466408.1), the next closest taxon was a strain of *F. nygamai* (PUF025 = HQ423219.1) with 99% similarity (788/794 bp). The *rPB2* sequence of CBS 483.94 and CBS 119850 (= KU604268) shared 99% similarity (785/791 bp) with *F. nygamai* (FRC M-7492 = KF466408.1) in GenBank, *G. fujikuroi* species complex (GFSC) with 98.74% similarity in FUSARIUM ID, and *F. nygamai* (CBS 749.97) with 98.74% similarity in *Fusarium* MLST. The different indication of the species complexes, either with *Gibberella* or with *Fusarium*, is due to the use of either the name of the sexual or the asexual morph, respectively; at present the name *Fusarium* is preferred over *Gibberella* and hence the same species complex is now known as FFSC.

**Fig. 1:**
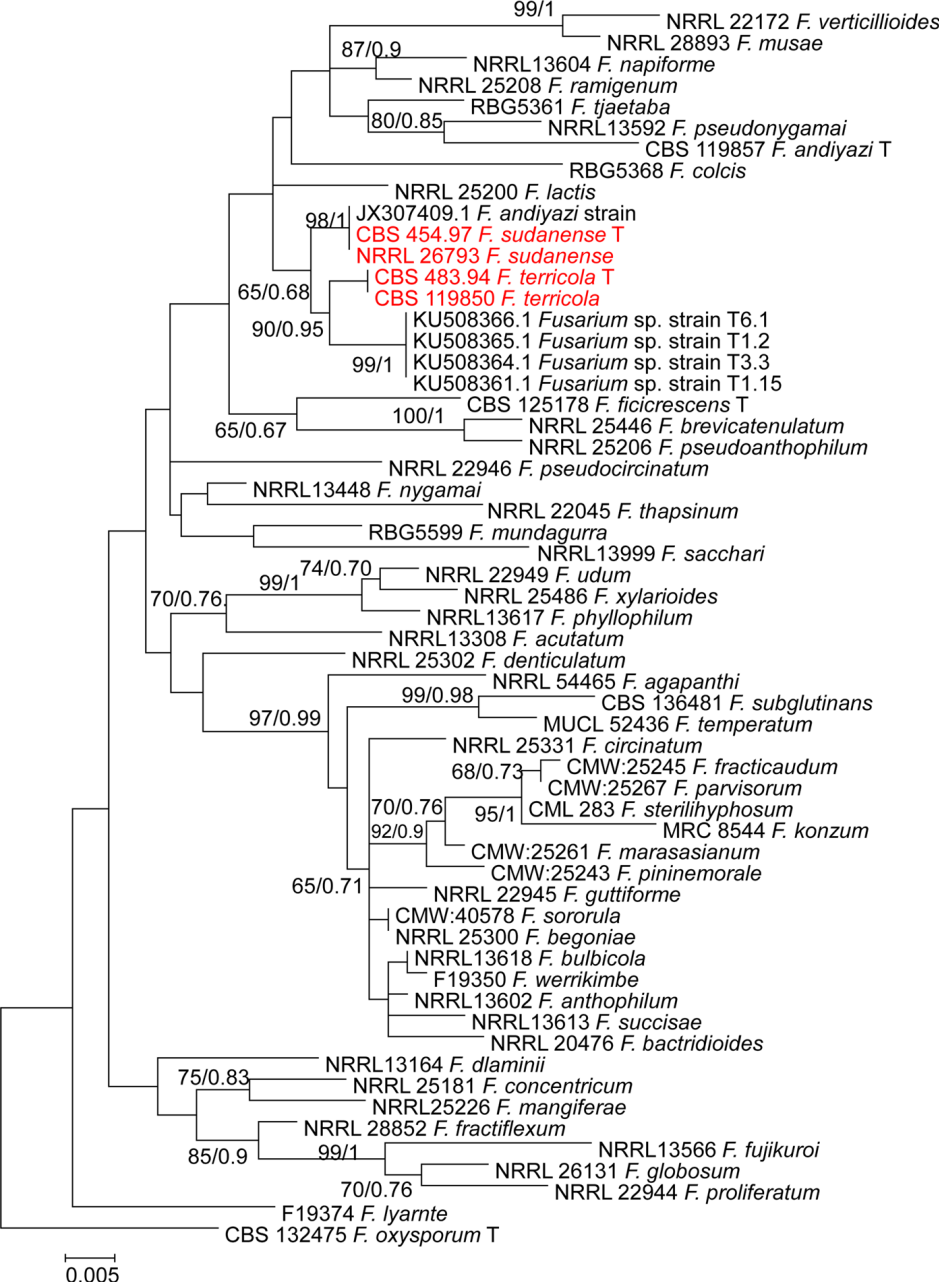
Phylogenetic tree generated by Bayesian inference (BI) and maximum likelihood (ML) trees from 58—*TEF1* sequences, 576 characters, 10,000,000 generations, 4 mcmc runs. Numbers on the branches are Bayesian posterior probabilities (PP), percentages of 1000 bootstrap-replications of MEGA6-maximum likelihood (PP/ML). The tree was rooted with the two strains *F. oxysporum* CBS 132475

**Fig. 2:**
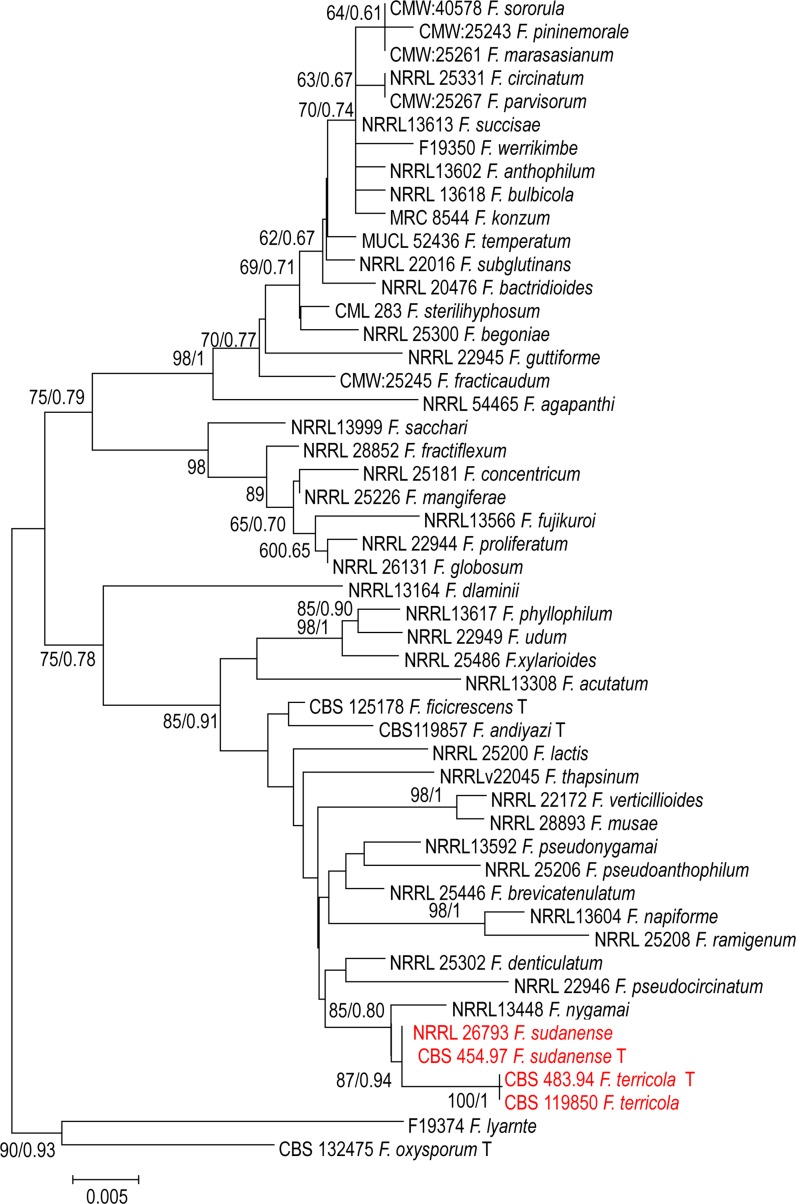
Phylogenetic tree generated by Bayesian inference (BI) and maximum likelihood (ML) trees from 50—*BT2* sequences, 500 characters, 10,000,000 generations, 4 mcmc runs. Numbers on the branches are Bayesian posterior probabilities (PP), percentages of 1000 bootstrap-replications of MEGA6-maximum likelihood (PP/ML). The tree was rooted with the two strains *F. oxysporum F. oxysporum* CBS 132475

**Fig. 3:**
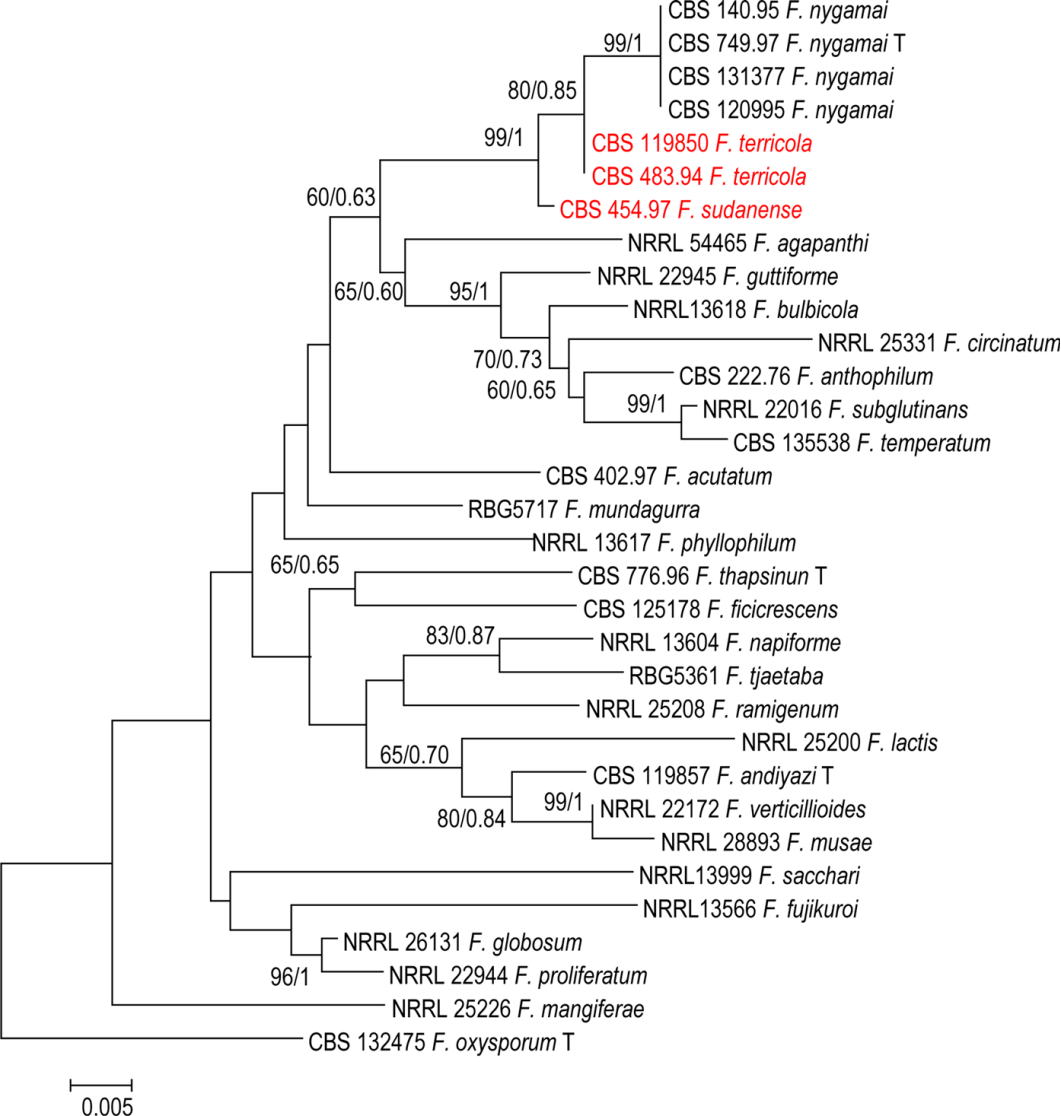
Phylogenetic tree generated by Bayesian inference (BI) and maximum likelihood (ML) trees from 32—*RPB2* sequences, 860 characters, 10,000,000 generations, 4 mcmc runs. Numbers on the branches are Bayesian posterior probabilities (PP), percentages of 1000 bootstrap-replications of MEGA6-maximum likelihood (PP/ML). The tree was rooted with the two strains *F. oxysporum* CBS 132475

For further understanding of relations between species, a phylogenetic tree was constructed for each locus separately, i.e. *TEF1*, *BT2*, and *rPB2*. In each single tree of *BT2*, *rPB2* and *TEF1* separately, strains CBS 119850 and CBS 483.94 from soil in Australia, and an additional strain CBS 454.97 from plant debris in Sudan were found to form a monophyletic clades supported by a high bootstrap values (Figs. 1, 2, 3).

The *TEF1* dataset comprising 58 sequences consisted of 53 taxa with 576 characters, from which 202 were variable, 111 parsimony-informative and 91 were singletons. Phylogenetic analyses of 50 sequences of *BT2* resolved the phylogenetic positions of the two novel taxa in relation to the currently recognised monophyletic species in the *F. fujikuroi* species complex used in the current analysis (Figs. 1, 2). The *BT2* dataset comprising 50 sequences consisted of 48 taxa with 500 characters, from which 129 were variable, 70 parsimony-informative and 58 were singletons. In our study, we were able to cover all taxa which have *rPB2* sequences retrieved from the GenBank. We used 32 sequences retrieved from GenBank representing 28 species of the *fujikuroi* complex. Ribosomal polymerase B2 (*rPB2*) is one of the most informative gene fragments and resolves taxonomy at or near the species-level in *Fusarium,* but its drawback is that fewer sequences are available in GenBank. The alignment of *rPB2* sequences had a length of 800 nucleotides when the outgroup was included; 175 were variable, 104 parsimony-informative and 71 were singletons.

**Fig. 4:**
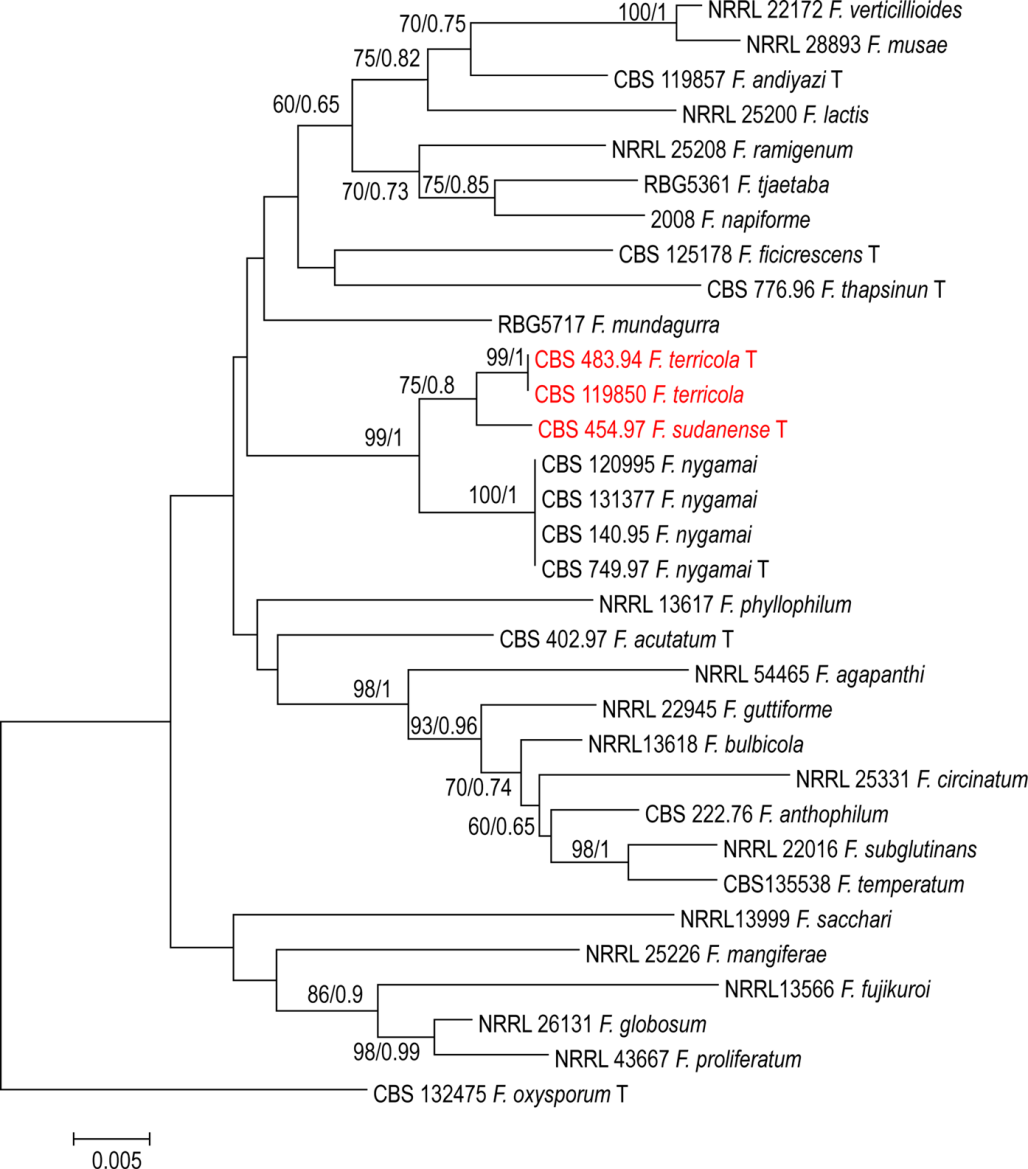
Phylogenetic tree generated by Bayesian inference (BI) and maximum likelihood (ML) trees from 32—*TEF1* + *RPB2* sequences, 1411 characters, 10,000,000 generations, 4 mcmc runs. Numbers on the branches are Bayesian posterior probabilities (PP), percentages of 1000 bootstrap-replications of MEGA6-maximum likelihood (PP/ML). The tree was rooted with the two strains *F. oxysporum* CBS 132475

The combined *TEF1* and *rPB2* alignment for 28 species consisted of 32 sequences each with 1411 characters; the ML/BI tree is shown in Fig. 4. The analysis indicated that the isolates (CBS 454.97) and (CBS 119850 and CBS 483.94) form distinct clades separated from other species of *fujikuroi* complex and these two clades have support (75% BS and 0.8 PP); for CBS 454.97, and (99% BS and 1 PP) support for (CBS 119850 and CBS 483.94 respectively) (Fig. 4). Bayesian and maximum likelihood phylogenetic trees constructed with *rPB2* sequences of available strains appeared well-resolved. All clades had statistical support between 60–100% and all species were well separated. Intraspecific polymorphism within the species clusters was observed with *BT2, TEF1* and *rPB2*. Overall topologies of the trees were similar to those described previously for the FFSC (Al-Hatmi et al. 2016c).

## Taxonomy

**Fig. 5:**
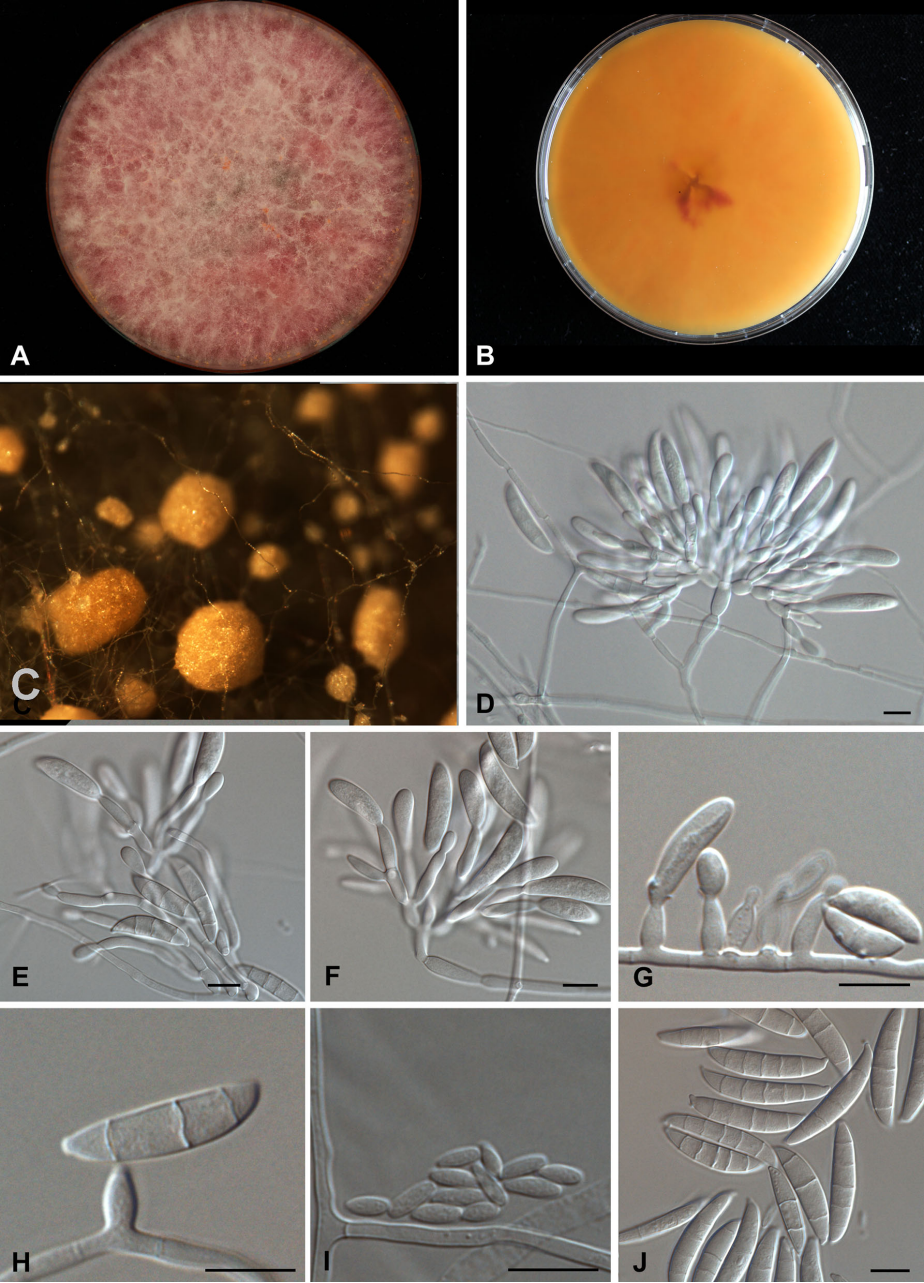
Morphological description of *Fusarium terricola* CBS 483.94. **a**–**b** Growth on MEA agar, front pinkish white, reverse orange; **c** Sporodochia; **e**–**f** Branching polyphialides. **g**–**h** Short monophialides; **i** Microconidia; **j** Septate macroconidia. *Scale bar* 10 µm

*Fusarium terricola* Al-Hatmi, S.A. Ahmed and de Hoog, sp. nov.—Fig.5. MycoBank MB 816188.

Etymology: terri cola means soil-loving, referring to the fungus’ apparently preferred habitat.

Holotype: dried specimen in herbarium CBS H-22548; living ex-type strain CBS 483.94, isolated from desert soil, Queensland, Australia.

Description based on CBS 483.94 on MEA and CLA growing in the dark at 27 °C after 7 days. Colonies growing rapidly, attaining 50 mm diam. Obverse aerial mycelium cottony, initially white and later becoming pinkish to purple on MEA (Fig. 5). Reverse pinkish-orange to darker purple. Sporodochia seen after 7 days of incubation as pale orange spots on pieces of carnation leaf placed on CLA. Sporulation on SNA starting early in aerial mycelium and later on agar surface. Aerial conidiophores in darkness mostly prostrate, simple to sparsely branched, but some erect and branching sympodially or verticillately, resulting in a complex tree-like morphology (Fig. 5d). Conidiophores 90–100 μm; conidiogenous cells are mostly polyphialidic. Conidia produced mostly on phialides formed directly on substrate hyphae (Fig. 5g, h). Monophialides 10.0–14.5 × 3–5 μm, ellipsoidal, tapered towards the apex with minute basal frill. Microconidia ovoidal, 5.7–4.2 × 1.8–2.4 μm. Macroconidia abundant, 24.0–31.9 × 5.6–6.0 μm, 2–5 septate, falcate, with a beaked apical cell and a foot-like basal cell (Fig. 5j). Chlamydospores absent.

**Fig. 6:**
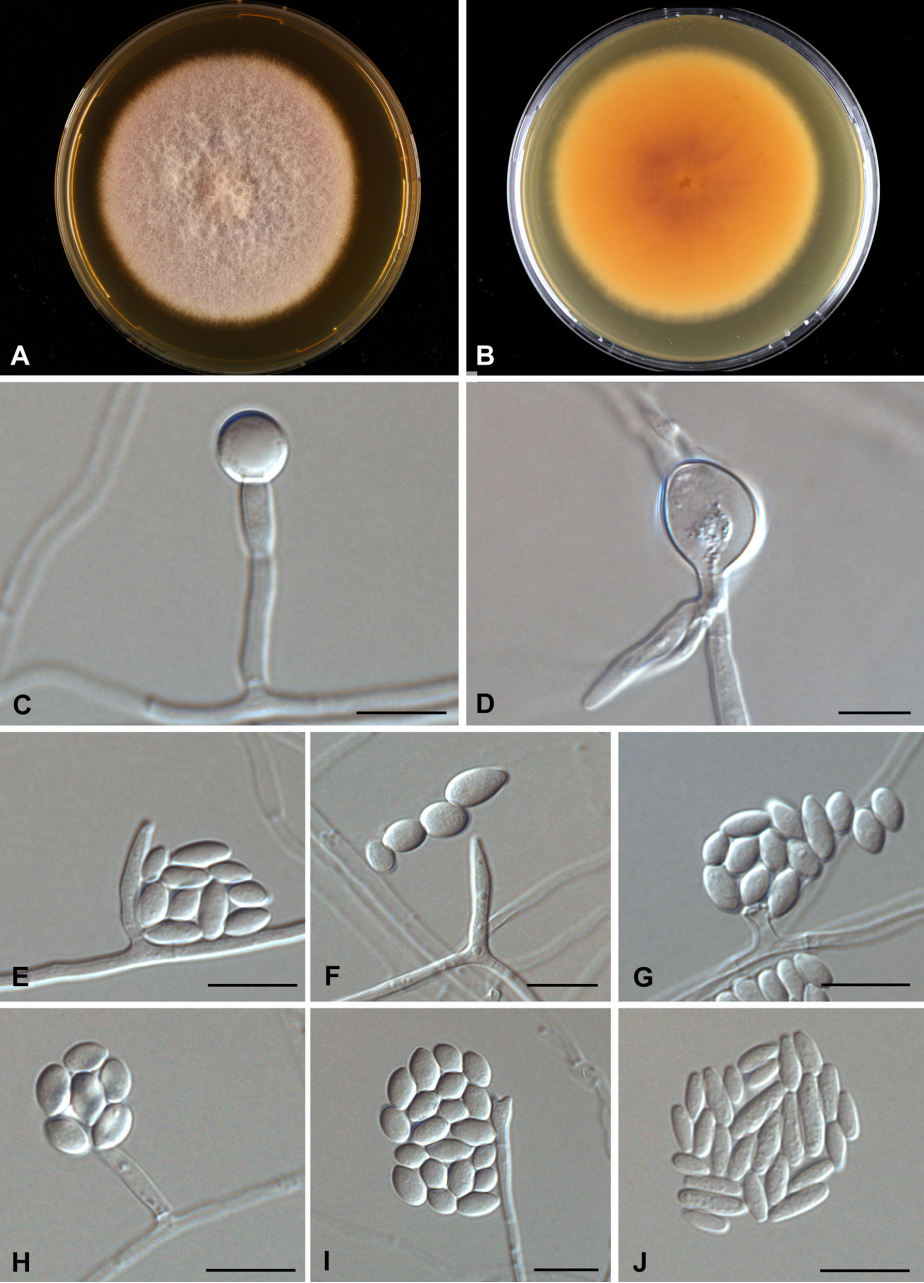
Morphological description of *Fusarium sudanense* CBS 454.97. **a**–**b** Growth on MEA agar, front pinkish white, reverse orange; **c**–**d** single, verrucose chlamydospore on the tip of hyphae; **e**–**i** Short monophialides with false head and microconidia; **j** Microconidia, abundant and ovoidal. *Scale bar* 10 µm

*Fusarium sudanense* S.A. Ahmed, Al-Hatmi and de Hoog, sp. nov.—Fig. 6. MycoBank MB 816189.

Etymology: named after the country of isolation, Sudan.

Holotype: dried specimen in herbarium CBS H-22547; living ex-type strain CBS 454.97, from plant debris (*Striga hermonthica*), Sudan.

Description based on CBS 454.97 on MEA and CLA growing in the dark at 27 °C after 7 days. Colonies expanding, attaining 45 mm diam. Aerial mycelium cottony, initially white and later becoming light pinkish, reverse pink-orange (Fig. 6a, b). Hyphae 1.9–2.9 μm, smooth-walled, hyaline, branched, septate. Conidiophores phialidic with mostly monophialides, rarely polyphialdes (Fig. 6g–i). Monophialides 13.0–17.4 × 2.0–3.0 μm, elongate-ampulliform or subcylindrical and tapered at the apex, or short ossiform, wider at the base. Microconidia abundant, subspherical or ovoidal, 3.5–10.5 × 2.7–1.7 μm (Fig. 6j) Macroconidia not seen. Chlamydospores appearing after 1 week of incubation, single or in chains, consisting of enlarged, thick-walled vegetative cells within hyphae (intercalary) or at hyphal tips (terminal), 8–13 μm diam (Fig. 6c, d).

Cardinal growth temperature tests showed that all cultures evaluated in this study had their optimal development at 27–33 °C, with growth abilities ranging between 18 °C the lowest temp tested and 40 °C as the highest. All strains were still able to grow at 37 °C, but not at 40 °C.

## Discussion

This study was initiated to characterize *Fusarium* strains held at the CBS reference collection at Utrecht, The Netherlands using polyphasic approaches. Phylogenetic analyses of a 3-gene dataset strongly supported the genealogical exclusivity of *F. terricola* and *F. sudanense* (Taylor et al. 2000). Both species received strong monophyletic bootstrap support in the individual analysis of each gene (Figs. 1, 2, 3) and combined (Fig. 4). Despite phylogenetic differences, *F. terricola* and *F. sudanense* isolates are morphologically similar to the remaining species in the *F. fujikuroi* species complex, however, there are several morphological difference between both species. The morphological description was based on two strains and therefore the phenotypic variability of the described species cannot be predicted. Morphological species concepts are regarded to be unreliable at the species level in *Fusarium* taxonomy (Al-Hatmi et al. 2016d). Diagnostic morphological characteristics between species are not easily observed due to intraspecific variation and because *Fusarium* species over longer phylogenetic distances may look very similar. The biological species concept in the genus is rudimentary due to lack of sexual recombination in several species groups and because the concept may be complicated by parasexuality, hybridization and horizontal gene transfer (Park 2013). For this reason genealogical concordance and absence of recombination between lineages is therefore mostly applied for species delimitation (Taylor et al. 2000).

To overcome possible problems due to phenotypic overlapping, we applied multigene phylogenies to recognize species boundaries. The *TEF1* alpha, is the recommended barcoding region for clinical *Fusarium* spp. (Stielow et al. 2015; Al-Hatmi et al. 2016c). The grouping of the *F. terricola* and *F. sudanense* was clear based on *TEF1* data. *Fusarium terricola* and *F. sudanense* were seen as a sister clade, closely related to undescribed species KU508366.1 *Fusarium* sp. strain T6.1 (Fig. 1). Additional *BT* and *rPB2* sequences data, however, significantly improved resolution and confirmed *F. terricola* and *F. sudanense* as two clades distinct from *F. fujikuroi complex,* closely related to *F. nygamai* (Figs. 2, 3). MLH-BI analyses of the *TEF1*-α, *BT* and *rPB2* loci strongly supported a sister group relationship between *F. terricola* and *F. sudanense* and maintained their status as independent evolutionary lineages (Figs. 1, 2, 3).

Based on the phylogenetic species concept, molecular diagnostics using available genetic marker sequences have played an important role in understanding the systematics of the *Fusarium* (Geiser et al. 2004; O’Donnell et al. 2010). The selected marker sequences *TEF1*, *BT* and *rPB2* still have limitations such as incongruent topologies among single gene trees and lack of resolution needed to distinguish species boundaries. For example, our *TEF1* tree (Fig. 1) shows different species (NRRL 25200, *F. lactis* and T6.1 *Fusarium* sp.) as being closest relatives of the proposed taxa, while *BT*, *rPB2* and the concatenated trees indicate *F. nygamai* as being closely related (Figs. 2, 3, 4). In the *Fusarium fujikuroi* complex several genes such as *TEF1*, *rPB2* and *BT* have been used in the construction of the species phylogeny due to their highly conserved regions and the reasonable degree of variation among multiple taxa. However, our results show incongruency among these genes. For example, the molecular phylogeny based on sequenced *TEF1* is incongruent with the *rPB2* and BT as a single gene. This might be due to some recombinations going on within the clade in *TEF1*.

A lack of concordance between molecular markers such as *TEF1*, *rPB2* and IGS within the *F. oxysporum* complex has been reported by O’Donnell et al. (2009). Incongruency between single gene phylogenies above species level can be caused by a combination of analytical and biological factors, the analytical factors including taxon sampling, outgroup selection, criteria of optimality, and modeling of sequence evolution in phylogeny construction (Rokas et al. 2003). As biological factors, some studies considered natural selection, recombination and genetic drift of *Fusarium* species (Rokas et al. 2003; Taylor et al. 1999).This might tell us that the *Fusarium* taxonomy has a fundamental flaw due to ongoing evolution and incomplete lineage sorting.

In the present study we characterized two novel *Fusarium* species recovered from soil and plant debris as *F. terricola* and *F. sudanense*. Further research is needed to determine the relation between opportunism on plants or on humans, because both species had an optimum growth around 27 °C and were still able to grow at 37 °C, but not at 40 °C. They thus potentially might be able to cause infections in humans and plants, but invasion of living organisms has as yet not been observed.

## References

[CR1] Al-Hatmi AM, Normand AC, van Diepeningen AD, Hendrickx M, de Hoog GS, Piarroux R (2015). Rapid species-level identification of opportunists in the *Fusarium fujikuroi* species complex using MALDI-TOF mass spectrometry. Future Microbiol.

[CR2] Al-Hatmi AM, Mirabolfathy M, Hagen F, Normand AC, Stielow JB, Karami-Osbo R, van Diepeningen AD, Meis JF, de Hoog GS (2016). DNA barcoding, MALDI-TOF and AFLP data support *Fusarium ficicrescens* as a distinct species within the *F. fujikuroi* species complex. Fungal Biol.

[CR3] Al-Hatmi AM, Van Den Ende AH, Stielow JB, Van Diepeningen AD, Seifert KA, McCormick W, Assabgui R, Gräfenhan T, De Hoog GS, Levesque CA (2016). Evaluation of two novel barcodes for species recognition of opportunistic pathogens in *Fusarium*. Fungal Biol.

[CR4] Al-Hatmi AM, Meis JF, de Hoog GS (2016). *Fusarium*: molecular diversity and intrinsic drug resistance. PLoS Pathog.

[CR5] Al-Hatmi AMS, Hagen F, Menken SBJ, Meis JF, de Hoog GS (2016). Global molecular epidemiology and genetic diversity of *Fusarium*, a significant emerging group of human opportunists, 1958-2015. Emerg Microbes Infect.

[CR6] Alves-Santos FM, Benito EP, Eslava AP, Díaz-Mínguez JM (1999). Genetic diversity of *Fusarium oxysporum* strains from common bean fields in Spain. Appl Environ Microbiol.

[CR7] de Nijs M, van Egmond HP, Rombouts FM, Notermans SHW (1997). Identification of hazardous *Fusarium* secondary metabolites occurring in food raw materials. J Food Saf.

[CR8] Edwards J, Auer D, de Alwis SK, Summerell B, Aoki T, Proctor R, Busman M, O’Donnell K (2016). *Fusarium agapanthi* sp. nov, a novel bikaverin and fusarubin-producing leaf and stem spot pathogen of *Agapanthus praecox* (African lily) from Australia and Italy. Mycologia.

[CR9] Fisher JD, Nadler A, Whitcher-Alagna S (1982). Recipient reactions to aid. Psychol Bull.

[CR10] Geiser DM, Jimenez-Gasco MD, Kang SC, Makalowska I, Veeraraghavan N, Ward TJ, Zhang N, Kuldau GA, O’Donnell K (2004). FUSARIUM-ID v. 1.0: a DNA sequence database for identifying *Fusarium*. Eur J Plant Pathol.

[CR11] Herron DA, Wingfield MJ, Wingfield BD, Rodas CA, Marincowitz S, Steenkamp ET (2015). Novel taxa in the *Fusarium fujikuroi* species complex from *Pinus* spp.. Stud Mycol.

[CR12] Huelsenbeck JP, Ronquist F (2001). MRBAYES: Bayesian inference of phylogenetic trees. Bioinformatics.

[CR13] Karim NFA, Mohd M, Nor NMIM, Zakaria L (2016). Saprophytic and potentially pathogenic *Fusarium* species from peat soil in Perak and Pahang. Trop Life Sci Res.

[CR14] Larget B, Simon DL (1999). Markov chain Monte Carlo algorithms for the Bayesian analysis of phylogenetic trees. Mol Biol Evol.

[CR15] Laurence MH, Walsh JL, Shuttleworth LA, Robinson DM, Johansen RM, Petrovic T, Vu TTH, Burgess LW, Summerell BA, Liew ECY (2015). Six novel species of *Fusarium* from natural ecosystems in Australia. Fungal Diver.

[CR16] Leslie JF, Summerell BA (2006). The *Fusarium* Laboratory Manual. Oxford: Blackwell Publishing Ltd, Ames. materials. J Food Saf.

[CR18] Nirenberg HI (1976). Untersuchungen über die morphologische und biologische Differenzierung in der *Fusarium* Sektion *Liseola*. Mitt Biol Bundesanst Land- u Forstw (Berlin-Dahlem).

[CR19] Nirenberg HI, O’Donnell K (1998). New *Fusarium* species and combinations within the *Gibberella fujikuroi* species complex. Mycologia.

[CR20] O’Donnell K, Nirenberg HI, Aoki T, Cigelnik E (2000). A multigene phylogeny of the *Gibberella fujikuroi* species complex: detection of additional phylogenetically distinct species. Mycoscience.

[CR66] O’Donnell K, Sarver BA, Brandt M, Chang DC, Noble-Wang J, Park BJ, Sutton DA, Benjamin L, Lindsleyn M, Padhye A, Geiser DM, Ward TJ (2007) Phylogenetic diversity and microsphere array-based genotyping of human pathogenic Fusaria, including isolates from the multistate contact lens-associated U.S. keratitis outbreaks of 2005 and 2006. J Clin Microbiol 45:2235–2248 10.1128/JCM.00533-07 PMC1933018 17507522

[CR22] O’Donnell K, Gueidan C, Sink S, Johnston PR, Crous PW, Glenn A, Riley R, Zitomer NC, Colyer P, Waalwijk C, Lee T, Moretti A, Kang S, Kim HS, Geiser DM, Juba JH, Baayen RP, Cromey MG, Bithell S, Sutton DA, Skovgaard K, Ploetz R, Corby Kistler H, Elliott M, Davis M, Sarver BA (2009). A two-locus DNA sequence database for typing plant and human pathogens within the *Fusarium oxysporum* species complex. Fungal Genet Biol.

[CR23] O’Donnell K, Sutton DA, Rinaldi MG, Sarver BA, Balajee SA, Schroers HJ, Summerbell RC, Robert VA, Crous PW, Zhang N, Aoki T, Jung K, Park J, Lee YH, Kang S, Park B, Geiser DM (2010). Internet-accessible DNA sequence database for identifying Fusaria from human and animal infections. J Clin Microbiol.

[CR77] O’Donnell K, Rooney AP, Proctor RH, Brown DW, McCormick SP, Ward TJ, Frandsen RJN, Lysøe E, Rehner SA, Aoki T, Robert VA, Crous PW, Kang S, Geiser DM (2013) RPB1 and RPB2 phylogeny supports an early cretaceous origin and a strongly supported clade comprising all agriculturally and medically important fusaria. Fungal Genet Biol 52:20–31 10.1016/j.fgb.2012.12.004 23357352

[CR21] O’Donnell K, Ward TJ, Robert VARG, Crous PW, Geiser DM, Kang S (2015). DNA sequence-based identification of *Fusarium*: current status and future directions. Phytoparasitica.

[CR24] O’Donnell K, Sutton DA, Wiederhold N, Robert VA, Crous PW, Geiser DM (2016). Veterinary fusarioses within the United States. J Clin Microbiol.

[CR25] Ordonez N, Seidl MF, Waalwijk C, Drenth A, Kilian A, Thomma BP, Ploetz RC, Kema GH (2015). Worse comes to worst: bananas and panama disease—when plant and pathogen clones meet. PLoS Pathog.

[CR26] Park B (2013) Cyber-infrastructure supporting fungal and oomycete phylogenetics and genomics. Ph.D. Dissertation, Pennsylvania State University

[CR78] Proctor RH, van Hove F, Susca A, Stea G, Busman M, van der Lee T, Waalwijk C, Moretti A, Ward TJ (2013) Birth, death and horizontal transfer of the fumonisin biosynthetic gene cluster during the evolutionary diversification of Fusarium. Mol Microbiol 90:290–306 10.1111/mmi.12362 23937442

[CR27] Reeb V, Lutzoni F, Roux C (2004). Contribution of RPB2 to multilocus phylogenetic studies of the euascomycetes (Pezizomycotina, Fungi) with special emphasis on the lichen-forming Acarosporaceae and evolution of polyspory. Mol Phylogenet Evol.

[CR28] Rep M, Meijer M, Houterman PM, van der Does HC, Cornelissen BJC (2005). *Fusarium oxysporum* evades I-3-mediated resistance without altering the matching avirulence gene. Mol Plant Microbe Interact.

[CR29] Rokas A, Williams BL, King N, Carroll SB (2003). Genome-scale approaches to resolving incongruence in molecular phylogenies. Nature.

[CR88] Scauflaire J, Gourgue M, Munaut F (2011) Fusarium temperatum sp. nov. from maize, an emergent species closely related to Fusarium subglutinans. Mycologia 103:586–597 10.3852/10-135 21186324

[CR30] Smith SN (2007). An overview of ecological and habitat aspects in the genus *Fusarium* with special emphasis on the soil borne pathogenic forms. Plant Pathol Bull.

[CR31] Stamatakis A (2006). RAxML-VI-HPC: maximum likelihood-based phylogenetic analyses with thousands of taxa and mixed models. Bioinformatics.

[CR32] Stamatakis A, Ludwig T, Meier H (2005). RAxML-III: a fast program for maximum likelihood-based inference of large phylogenetic trees. Bioinformatics.

[CR33] Stielow B, Lévesque CA, Seifert KA, Meyer W, Irinyi L, Smits D, Renfurm R, Verkley GJM, Groenewald M, Chaduli D, Lomascolo A, Welti S, Lesage-Meessen L, Al-Hatmi AMS, Damm U, Yilmaz N, Houbraken J, Lombard L, Quaedvlieg W, Binder M, Vaas LAI, Vu D, Yurkov A, Begerow D, Roehl O, Guerreiro M, Fonseca A, Samerpitak K, van Diepeningen A, Dolatabadi S, Moreno L, Casaregola S, Mallet S, Jacques N, Roscini L, Egidi E, Bizet C, Garcia-Hermoso D, Martín-Esteban MP, Deng S, Groenewald JZ, Boekhout T, de Beer ZW, Barnes I, Duong T, Wingfield M, de Hoog GS, Crous PW, Schoch C, Lewis CT, Hambleton S, Moussa TAA, Al-Zahrani HS, Almaghrabi OA, Louis-Seize G, Assabgui R, McCormick W, Omer G, Dukik K, Cardinali G, Eberhardt U, de Vries M, Robert V (2015). One fungus, which genes? Assessing primers for potential universal secondary DNA barcodes. Persoonia.

[CR34] Summerell BA, Laurence MH, Liew ECY, Leslie JF (2010). Biogeography and phylogeography of *Fusarium*: a review. Fungal Divers.

[CR35] Taylor J, Jacobson D, Fisher M (1999). The evolution of asexual fungi: reproduction, speciation and classification. Annu Rev Phytopathol.

[CR36] Taylor JW, Jacobson DJ, Kroken S, Kasuga T, Geiser DM, Hibbett DS, Fisher MC (2000). Phylogenetic species recognition and species concepts in fungi. Fungal Genet Biol.

[CR89] Triest D, Stubbe D, De Cremer K, Piérard D, Detandt M, Hendrickx M (2015) Banana infecting fungus, Fusarium musae, is also an opportunistic human pathogen: Are bananas potential carriers and source of fusariosis? Mycologia 107:46–53 10.3852/14-174 25361833

[CR99] van Hove F, Waalwijk C, Logrieco A, Munaut F, Moretti A (2011) Gibberella musae (Fusarium musae) sp. nov., a recently discovered species from banana is sister to F. verticillioides. Mycologia 103:570–585 10.3852/10-038 21177490

[CR37] Wakelin SA, Macdonald LM, Rogers SL, Gregg AL, Bolger TP, Baldock JA (2008). Habitat selective factors influencing the structural composition and functional capacity of microbial communities in agricultural soils. Soil Biol Biochem.

[CR38] Watanabe M (2013). Molecular phylogeny and identification of *Fusarium* species based on nucleotide sequences. Mycotoxins.

